# Supplementary material for: A fault-intrusion-tolerant system and deadline-aware algorithm for scheduling scientific workflow in the cloud
Source: PeerJ Comput Sci. 2021 Nov 2;7:e747. doi: 10.7717/peerj-cs.747 (PMC8576554; doi:10.7717/peerj-cs.747)
Supplement: Supplemental Information 6 [file peerj-cs-07-747-s006.zip › Myproject1.1/docs/org/cloudbus/cloudsim/power/PowerVmAllocationPolicyAbstract.html]

PowerVmAllocationPolicyAbstract


JavaScript is disabled on your browser.


- Overview
- Package
- Class
- Use
- Tree
- Deprecated
- Index
- Help

- Prev Class
- Next Class

- Frames
- No Frames

- All Classes

- Summary:
- Nested |
- Field |
- Constr |
- Method

- Detail:
- Field |
- Constr |
- Method


org.cloudbus.cloudsim.power

## Class PowerVmAllocationPolicyAbstract

- java.lang.Object
- - org.cloudbus.cloudsim.VmAllocationPolicy
  - - org.cloudbus.cloudsim.power.PowerVmAllocationPolicyAbstract

- Direct Known Subclasses:
  :   PowerVmAllocationPolicyMigrationAbstract, PowerVmAllocationPolicySimple

  ---

    

  ```
  public abstract class PowerVmAllocationPolicyAbstract
  extends VmAllocationPolicy
  ```

  The class of an abstract power-aware VM allocation policy.
  If you are using any algorithms, policies or workload included in the power package, please cite
  the following paper:
  Anton Beloglazov, and Rajkumar Buyya, "Optimal Online Deterministic Algorithms and Adaptive
  Heuristics for Energy and Performance Efficient Dynamic Consolidation of Virtual Machines in
  Cloud Data Centers", Concurrency and Computation: Practice and Experience (CCPE), Volume 24,
  Issue 13, Pages: 1397-1420, John Wiley & Sons, Ltd, New York, USA, 2012

  Since:
  :   CloudSim Toolkit 3.0

- - ### Constructor Summary

    Constructors

    Constructor and Description

    `PowerVmAllocationPolicyAbstract(java.util.List<? extends Host> list)`

    Instantiates a new power vm allocation policy abstract.
  - ### Method Summary

    Methods

    | Modifier and Type | Method and Description |
    | --- | --- |
    | `boolean` | `allocateHostForVm(Vm vm)` Allocates a host for a given VM. |
    | `boolean` | `allocateHostForVm(Vm vm, Host host)` Allocates a specified host for a given VM. |
    | `void` | `deallocateHostForVm(Vm vm)` Releases the host used by a VM. |
    | `PowerHost` | `findHostForVm(Vm vm)` Find host for vm. |
    | `Host` | `getHost(int vmId, int userId)` Get the host that is executing the given VM belonging to the given user. |
    | `Host` | `getHost(Vm vm)` Get the host that is executing the given VM belonging to the given user. |
    | `java.util.Map<java.lang.String,Host>` | `getVmTable()` Gets the vm table. |

    - ### Methods inherited from class org.cloudbus.cloudsim.VmAllocationPolicy

      `getHostList, optimizeAllocation, setHostList`
    - ### Methods inherited from class java.lang.Object

      `clone, equals, finalize, getClass, hashCode, notify, notifyAll, toString, wait, wait, wait`

- - ### Constructor Detail


    - #### PowerVmAllocationPolicyAbstract

      ```
      public PowerVmAllocationPolicyAbstract(java.util.List<? extends Host> list)
      ```

      Instantiates a new power vm allocation policy abstract.

      Parameters:
      :   `list` - the list
  - ### Method Detail


    - #### allocateHostForVm

      ```
      public boolean allocateHostForVm(Vm vm)
      ```

      **Description copied from class: `VmAllocationPolicy`**

      Allocates a host for a given VM. The host to be allocated is the one that was already
      reserved.

      **Specified by:**
      :   `allocateHostForVm` in class `VmAllocationPolicy`

      Parameters:
      :   `vm` - virtual machine which the host is reserved to

      Returns:
      :   $true if the host could be allocated; $false otherwise


    - #### allocateHostForVm

      ```
      public boolean allocateHostForVm(Vm vm,
                              Host host)
      ```

      **Description copied from class: `VmAllocationPolicy`**

      Allocates a specified host for a given VM.

      **Specified by:**
      :   `allocateHostForVm` in class `VmAllocationPolicy`

      Parameters:
      :   `vm` - virtual machine which the host is reserved to

      Returns:
      :   $true if the host could be allocated; $false otherwise


    - #### findHostForVm

      ```
      public PowerHost findHostForVm(Vm vm)
      ```

      Find host for vm.

      Parameters:
      :   `vm` - the vm

      Returns:
      :   the power host


    - #### deallocateHostForVm

      ```
      public void deallocateHostForVm(Vm vm)
      ```

      **Description copied from class: `VmAllocationPolicy`**

      Releases the host used by a VM.

      **Specified by:**
      :   `deallocateHostForVm` in class `VmAllocationPolicy`

      Parameters:
      :   `vm` - the vm


    - #### getHost

      ```
      public Host getHost(Vm vm)
      ```

      **Description copied from class: `VmAllocationPolicy`**

      Get the host that is executing the given VM belonging to the given user.

      **Specified by:**
      :   `getHost` in class `VmAllocationPolicy`

      Parameters:
      :   `vm` - the vm

      Returns:
      :   the Host with the given vmID and userID; $null if not found


    - #### getHost

      ```
      public Host getHost(int vmId,
                 int userId)
      ```

      **Description copied from class: `VmAllocationPolicy`**

      Get the host that is executing the given VM belonging to the given user.

      **Specified by:**
      :   `getHost` in class `VmAllocationPolicy`

      Parameters:
      :   `vmId` - the vm id
      :   `userId` - the user id

      Returns:
      :   the Host with the given vmID and userID; $null if not found


    - #### getVmTable

      ```
      public java.util.Map<java.lang.String,Host> getVmTable()
      ```

      Gets the vm table.

      Returns:
      :   the vm table


- Overview
- Package
- Class
- Use
- Tree
- Deprecated
- Index
- Help

- Prev Class
- Next Class

- Frames
- No Frames

- All Classes

- Summary:
- Nested |
- Field |
- Constr |
- Method

- Detail:
- Field |
- Constr |
- Method
